# Volunteer altruistic behaviour in terms of disaster threat type

**DOI:** 10.4102/jamba.v15i1.1478

**Published:** 2023-11-14

**Authors:** Nevi K. Arianti, Koentjoro Koentjoro

**Affiliations:** 1 Faculty of Psychology, Universitas Gadjah Mada, Yogyakarta, Indonesia

**Keywords:** altruistic behaviour, community-based volunteers, types of disasters, *gotong royong*, disaster risk reduction

## Abstract

The altruistic behaviour of community-based volunteers in disaster situations determines the success rate of rescue achievements. The purpose of this study is to determine whether there are differences in the altruistic behaviour of volunteers in different types of disaster event threats, namely, landslides, volcanic eruptions, droughts and tsunamis in Yogyakarta. A quantitative study was conducted to answer the research question. The study involved 292 disaster volunteer participants, using the survey method. Hypothesis testing in this study used statistical methods in the form of one-way analysis of variance (ANOVA). The scores used were factor scores obtained from the results of confirmatory factor analysis (CFA) analysis. The data were analysed using jamovi software version 2.3.18. The results of the one-way ANOVA analysis showed no difference in altruism in the types of disaster threats of landslides, volcanic eruptions, droughts and tsunamis in Yogyakarta.

**Contribution:**

The results of this study can be considered as supporting information in developing programmes by supporting the altruistic behaviour of community-based disaster volunteers. The sincerity of community-based disaster volunteers in the form of altruistic behaviour is not influenced by the type of disaster threat. One form of collective altruistic behaviour that is important and prominent in Indonesian culture is *gotong royong. Gotong royong* is a joint activity of helping each other without being paid, which is local wisdom in Indonesia. It is therefore important for policymakers to preserve local wisdom values such as *gotong royong* in disaster risk reduction programmes across different types of disaster threats.

## Introduction

This article aims to find out whether there are differences in helping behaviour, especially the altruistic behaviour of community-based volunteers in disaster-prone areas that have different types of disaster threats. What is meant by volunteer altruistic behaviour in this study is the behaviour of volunteers in providing assistance to others (before, during and after a disaster) with the aim of the welfare of others or the community. Indonesia is a vast disaster-prone area. Various types of disasters exist so that it is often referred to as a ‘disaster supermarket’ (National Disaster Management Agency). Disasters that hit Indonesia are generally caused by hydrometeorology, floods, landslides and tornadoes, which dominate natural disasters that have occurred over the past decade (Yulianto et al. 2021). Not only natural disasters but social disasters are also the next threat if the governance of disaster relief handling is not improved continuously along with the dynamics on the ground (Rodríguez, Donner & Trainor 2018). Social disasters can be prevented by simultaneously improving disaster risk reduction governance. Community-based volunteers are the frontline for life safety and disaster risk reduction for communities in disaster-prone areas because they live in disaster-prone areas.

Zubenko and Capozzoli (2002) state that there are six phases in the disaster response cycle experienced by disaster survivor communities in general. The fifth phase is the ‘disillusionment’ phase, which occurs within a few months to a year after the disaster. Looking at the duration of the ‘disillusionment’ phase, which is mentioned in the period of several months to 1 year after the disaster, in the review of the post-disaster stress cycle approach, this is what happens to disaster survivors, namely post-disaster stress with signs of various dissatisfaction and disappointment with what happened. Interestingly, this phase, often referred to as the ‘second disaster’, is commonly a social disaster, where disaster survivor communities experience negative emotions again related to the disaster caused by, for example, the delay in assistance, the lack of distribution of assistance and the end of the assistance programme by a non-governmental or official agency. The sense of community decreases along with the natural process of the community to be able to come to terms with the limitations of existing assistance according to reality and start building itself according to its existing capacity (Kumala 2021). In a preliminary study conducted by researchers in early May 2022, community-based volunteers consist of formal and informal volunteers. Formal volunteers are registered in the local volunteer community, while informal volunteers do not belong to any community because they tend to move according to their personal choice of time and availability. Informal volunteers often do not stay with the disaster survivor community, they just come and go as needed. However, there are also informal volunteers who live in disaster survivor communities, because there is no official community that accommodates them. These volunteers are not bound by a rule or structure so they try to see every opportunity that exists and relatively do not experience the disappointment phase. Whereas formal volunteers will actually feel the disappointment phase more because they are structurally commanded and have a programme of activities to do. In reviewing the definition of volunteers based on the findings of Utomo and Minza (2016) in their research in the Merapi volcano disaster area, the term informal volunteer is synonymous with spontaneous volunteer where this type of volunteer is a volunteer who is not bound to an organisation and moves based on the urge to help spontaneously. While the term formal volunteer in the author’s findings is synonymous with the term organised volunteer, where the volunteer is registered as a member of a community which happens to be a community living in disaster-prone areas. It is interesting to see how the altruistic behaviour of informal and formal community-based volunteers move together in this situation.

Research conducted by Monllor, Pavez and Pareti (2020) on informal volunteers shows that disaster situations provide opportunities for those who follow the logic of efficiency by utilising the situation and taking advantage to develop disaster relief businesses, for example, the phenomenon of a charger rental business with diesel electricity in the Bantul area, Yogyakarta during the 2006 earthquake disaster. In terms of entrepreneurial law and economic reasons, this behaviour cannot be faulted. But in terms of Indonesian culture, such business behaviour is unethical despite the market need. *Gotong royong* is a more appropriate answer to fulfil the needs of disaster survivor communities. The definition of *gotong royong* according to KBBI (2002) is the activity of working together or helping each other. Tashadi (1982) states that *gotong royong* is a form of cooperation carried out through the mobilisation of energy to achieve a certain goal. Meanwhile, Koentjaraningrat (1974) says that *gotong royong* is the unpaid mobilisation of human labour for a project or work that is beneficial to the public or useful for development. *Gotong royong* behaviour is divided into two. The first is helping behaviour in agricultural activities, households, parties, celebrations and disasters. The second type is *gotong royong kerjabakti*, which is for public purposes (Koentjaraningrat 1987). It can be in the form of material (money and goods), physical labour and mental-spiritual (contribution of thoughts-constructive advice and prayer support).

Community-based volunteers are the keys to the effectiveness of *gotong royong* movement in disaster situations by taking action together for the common good. It is in this mutual aid behaviour that altruistic behaviour is embedded. Strong community bonds will be able to reduce the likelihood of long-term psychological impacts of disasters. Communities that care for each other will be better in overcoming their difficult times collectively than people who live in individualistic environments (Kumala 2021). Baron and Byrne (2005) state that helping behaviour is an action that provides direct benefits to other individuals, without providing benefits to the individual providing the help. Mutual aid behaviour is an altruistic behaviour that arises from trust and cooperation.

Schein (2009) further states that helping is commonly found in everyday life. In the context of disaster-prone areas, the daily tradition of *gotong royong* as an altruistic behaviour of community-based volunteers in the pre-disaster period becomes the community’s trust capital for more synergy during disasters and post-disaster recovery periods. Schein (2009) also argues that helping behaviour is a complex thing that can occur in all situations. Because of this complexity, the help provided can be beneficial, but it can also be perceived as having no impact on the individual being helped. In this case, it is understandable that sometimes even in *gotong royong*, which is actually altruistic, there are shortcomings and dynamics. That is why mutual aid activities whose purpose is to reduce the burden are sometimes not optimally beneficial or even increase the burden on disaster survivors (Rogstadius et al. 2013). It can be explained by Laguna et al. (2020) who state that the perception of the helper affects the act of helping. The perception that other individuals need certain help encourages a person to be more willing to help even without being asked, which is not necessarily the help needed by the survivor (Laguna et al. 2020). When the perceptions of the helper and the helped are different, it is not surprising that the form of help provided is different from the needs of disaster survivors.

Bierhoff (in Marjanovic, Sruthers & Greenglass 2012) argues that helping is an action aimed at the welfare of others driven by selfish and altruistic motives. Amato (1990) distinguishes the forms of helping behaviour into two, namely spontaneous helping and planned helping. Planned helping is divided into: (1) formal planned helping which is helping behaviour aimed at helping individuals or groups through agencies or organisations and (2) informal planned helping which is helping behaviour aimed at people who are already known and have closeness such as friends or family members. Spontaneous helping is helping behaviour aimed at strangers who we do not know. This behaviour occurs suddenly or spontaneously and is not planned in advance.

The altruistic behaviour of community-based volunteers is classified as formal planned helping and also informal planned helping. Utomo and Minza (2016) in their research show that it is possible to change the form of helping behaviour from spontaneous helping behaviour to planned helping behaviour. In Indonesia, organised volunteers have more potential and opportunity to get assistance and capacity-building support from the government than unorganised volunteers. The legality of volunteer organisations is required to access various financial and capacity-building support. However, because of the wide coverage of disaster areas and the large number of people in Indonesia, along with the limited government budget, the capacity-building approach according to local culture is more likely to be synergised with capacity-building from outside the community.

Kassin, Fein and Markus (2014) present three aspects of helping behaviour, namely rewards, empathy, and altruism and egoism as a unit. (1) Reward is the reward that individuals receive when they have done a help. One of the individual’s help reasons is to obtain psychological and material rewards. Individuals often feel happy when helping other individuals. (2) Empathy is the feeling of understanding an individual’s perspective either directly or indirectly and feeling sympathy or love from that individual. (3) Altruism and egoism are two things that cause individuals to perform helping behaviour. Individuals who help based on altruism have a desire to improve the welfare of other individuals and do not think about the rewards they will receive. Individuals can also perform helping behaviour because of egoism, which is the desire to improve their own well-being. Individuals perform helping behaviour to increase positive feelings within themselves.

Realising that the disaster management process is not easy and simple, multi-stakeholder cooperation is a necessity. Various government agencies and non-government agencies work together to help disaster victims from pre-disaster, during disaster and post-disaster stages under the coordination of the Regional Disaster Management Agency (BPBD) or the National Disaster Management Agency (BNPB) based on the status of the disaster. Community-based volunteers, whether under the assistance of BPBD, provincial search and rescue (SAR), social services or independent communities, work together. Elements of village officials, village guidance officers (Babinsa), community security and order officers (Babinkamtibmas), and religious and traditional leaders were members of the community-based volunteer community in this study. The heterogeneity approach of the various volunteers’ background who become members of the community is seen to answer the needs of disaster management that must be supported by various parties. Homogeneous community-based volunteers often experience various obstacles in carrying out their duties. Among them are limited competence, the number of human resources, as well as facilities and infrastructure. Koentjoro and Andayani (2006) state that the helping behaviour of disaster volunteers is important based on four Community Development approaches, namely: (1) awareness that helping is not an easy job; (2) helping must optimise the role of the person being helped; (3) helping must empower the person being helped and (4) helping must prosper the person being helped, not causing dependence on disaster survivors. Therefore, to answer the needs of disaster survivors, appropriate helping actions are needed, supported by multi-stakeholder cooperation.

Mutual aid is a form of multi-stakeholder psychosocial support in disaster situations, as psychosocial support is the entire process of channelling assistance to disaster survivors (Rodríguez et al. 2018). Psychosocial support is referred to as the entire assistance process because it includes pre-disaster support, disaster support and post-disaster support. What is meant as mental health strengthening assistance, economic assistance such as strengthening livelihoods, physical assistance and information assistance are examples of forms or types of psychosocial support provided.

Chaplin (2006) states that psychosocial is a psychological aspect that explains social relationships include various aspects of psychology. Meanwhile, Baron and Byrne (2005) suggest that psychosocial is knowledge that seeks to understand the causes of individual behaviour and thinking in the context of social situations. According to Kemenpppa, psychosocial is a dynamic relationship between the psychological and social aspects of a person. Psychosocial support is any form of support from local or external parties that aims to maintain or promote psychosocial well-being and/or prevent or overcome mental disorders. Thus it can be said that psychosocial support is a form of assistance provided to individuals who experience psychological disorders because of disasters. Psychosocial support is carried out continuously. The psychological and social aspects mutually influence each other in the individual’s environment. Smet (1994) suggests four dimensions of psychosocial support, namely: (1) Emotional support, in the form of expressions of feelings of care, empathy and concern for other individuals; (2) Appreciative support, in the form of positive expressions as a form of respect for other individuals; (3) Instrumental support, in the form of direct material assistance and (4) Informative support, in the form of providing advice, suggestions or feedback to individuals.

Community-based volunteers, because of their unique position at the frontline of disaster-prone areas and characterised by mutual cooperation, are clearly the spearhead of the success of disaster management programmes. Trained and equipped volunteers are very effective in coping with disasters during emergencies. However, with the vast territory, natural morphology with unreachable transportation access and diverse cultures in Indonesia as well as the number of trained volunteers and the limited government budget in Indonesia, the approach of strengthening community-based disaster volunteers is a more reasonable necessity for Indonesia’s disaster governance. Disaster-based volunteers living in disaster areas are important targets in realising a trained and equipped community of volunteers with standardised equipment. In order to provide effective assistance, it is necessary to examine the helping behaviour of these community-based volunteers, especially in their altruistic-cooperative behaviour, because different types of disaster threats require different treatments. Thus the question raised in this study is whether there are differences in the altruistic behaviour of community-based volunteers with different types of disaster threats.

## Research methods and design

### Respondents

The respondents in this study were community-based volunteers experienced in natural disasters in Yogyakarta Special Region with a total of 292 respondents, aged between 20 and 61 years (*M* = 43.74; standard deviation [SD] = 8.95). They are community-based volunteers, who live and work in landslide, volcanic, drought, or tsunami hazard areas, spread across various communities in Kulon Progo, Sleman and Gunung Kidul districts. This technique is guided by the principle of convenience based on the suitability of subjects according to population criteria (Priyono 2016). What is meant by the principle of convenience based on the suitability of the subject according to the population criteria is the accuracy of the subject criteria with the population criteria. Table 1 shows the distribution of respondents based on sex, education level and duration or length of volunteering and type of natural disaster threat.

**TABLE 1:** Distribution of respondents.

Criteria	*n*	%
**Sex**		
Male	208	28.77
Female	84	71.23
**Education level**		
Elementary school	42	14.38
Junior high school	80	27.4
Senior high school	147	50.34
University	22	7.53
Abstain	1	0.34
**Length of volunteering**		
2–5 years	97	33.22
5–10 years	128	43.84
> 10 years	67	22.95
**Types of natural disasters**		
Landslides	149	51.03
Tsunamis	8	2.74
Volcanic eruptions	110	37.67
Droughts	25	8.56

*Source:* Arianti, N.K. & Koentjoro, K., 2023, ‘Volunteer altruistic behaviour in terms of disaster threat type’, *Jàmbá: Journal of Disaster Risk Studies* 15(1), a1478. https://doi.org/10.4102/jamba.v15i1.1478

### Research instruments

The altruism instrument was compiled by researchers based on Bierhoff’s theory (in Marjanovic et al. 2012) which consists of six items. The altruism scale in this study was based on aspects of helping to prosper others altruistically. This instrument was structured using a Likert scale with five response options (1 = Very unsuitable to 5 = very suitable). The reliability score estimation using Cronbach’s alpha resulted in a value of 0.66 (95% confidence interval [CI] = 0.60–0.72). The CFA analysis resulted in a model accuracy index value of root mean square error of approximation (RMSEA) = 0.090 (90% CI = 0.056–0.126) *p* RMSEA < 0.05; CFI = 0.840; Tucker-Lewis index (TLI) = 0.733; standardised root mean square residual (SRMR) = 0.052; with a standardised factor load of 0.42–0.56.

### Research procedure

The procedures in this study included the process of developing measuring instruments which were then reviewed by two lecturers, two disaster volunteer practitioners and the research ethics commission of the Faculty of Psychology, Gadjah Mada University to fulfil ethical standards in accordance with the context of variable measurement and the psychological research code of ethics. Data collection was carried out directly in the field or disaster-prone areas by first contacting key figures, namely BPBD officials and community leaders as well as coordinators of volunteer communities in three districts, namely in Kulon Progo, Sleman and Gunung Kidul.

### Data analysis

Hypothesis testing in this study used a statistical method in the form of one-way ANOVA. The score used was the factor score (DiStefano, Zhu & Mîndrilã 2009) obtained from the CFA analysis results. The data were analysed using jamovi software version 2.3.18 (The Jamovi Project 2022).

## Results

The homogeneity test using Levene’s test showed that the data variances were homogeneously distributed (*F*[3, 288] = 1.87; *p* = 0.14). Furthermore, the results of one-way ANOVA analysis showed no difference in altruism in terms of the type of natural disaster (*F*[3, 288] = 0.72; *p* = 0.54). Table 2 shows the categorisation of the level of altruism in respondents. Most respondents were in the medium level altruistic category (62.67%) followed by the high-level altruistic category (29.45%) and then the low-level altruistic category (7.88%). Respondents from the type of landslide disaster threat had a moderate category (29.45%), a high category (16.44%) and a low category (5.14%). While respondents with the type of Tsunami disaster threat had a moderate altruistic category (1.71%) and a high level of altruistic category (1.03%). Respondents with the type of volcano disaster threat were in the medium altruistic category (25.68%), high altruistic category (10.27%) and low altruistic category (1.71%). For respondents with the type of drought disaster threat, they were in the medium altruistic category (5.82%), the high altruistic category (1.71%) and the low category (1.03%).

**TABLE 2:** Altruism level categorisation.

Level of altruism category	Low		Medium		High	
	*n*	%	*n*	%	*n*	%
All respondents	23	7.88	183	62.67	86	29.45
**Types of disasters**	15	5.14	86	29.45	-	-
Landslides	-	-	5	1.71	48	16.44
Tsunamis	5	1.71	75	25.68	3	1.03
Volcanic eruptions	3	1.03	17	5.82	30	10.27
Droughts	-	-	-	-	5	1.71

*Source:* Arianti, N.K. & Koentjoro, K., 2023, ‘Volunteer altruistic behaviour in terms of disaster threat type’, *Jàmbá: Journal of Disaster Risk Studies* 15(1), a1478. https://doi.org/10.4102/jamba.v15i1.1478

## Discussion

This study shows that there is no difference in altruistic behaviour for community-based volunteers in disaster-prone areas with different types of disaster threats (*F*[3, 288] = 0.72; *p* = 0.54). The type of disaster threat, namely, landslides, volcanic eruptions, tsunamis and droughts, does not affect the sincerity of community-based volunteers in providing assistance according to their capacity. Community-based volunteers who live and live together in disaster-prone areas, experience firsthand, and see difficult situations in daily disaster conditions in the field. This finding is in line with the theory put forward by Silva, Marks and Cherry (2009) where individuals will be more easily moved to help after seeing situations that occur in the field, especially when there is a tradition of reciprocal altruism in the community, namely a culture of mutual cooperation in everyday life. It even starts when volunteers and residents provide assistance to each other in the community in the pre-disaster phase. The tradition of reciprocal altruism has an impact in disaster situations, where the feeling of attachment that exists gives its own impetus to help in disaster situations with different types of disasters.

Baron and Byrne (2005) argue that helping behaviour is an action that provides direct benefits to other individuals, without providing benefits to the individual who provides help. In this study, the altruistic behaviour of community-based volunteers refers more to the theory of Kassin et al. (2014) when there is sincerity in mutual cooperation behaviour, there is another side of the coin that accompanies it, namely receiving mutual benefits because of the helping behaviour. The benefit is at least a feeling of being valued and needed (there is a reward). Kassin et al.’s (2014) theory of helping behaviour aspects explains one important aspect of altruism and egoism in addition to the reward. The three experts believe that altruism and egoism are the two causes of individuals performing helping behaviour. Individuals who help in *gotong royong* are based on altruism, which has a desire to improve the welfare of other individuals and does not think about the rewards that will be received, but at the same time is also driven by the desire for self-satisfaction for successfully helping. It is also possible for community-based volunteers to perform helping behaviour because of egoism, which is the desire to increase positive feelings within themselves such as self-satisfaction for being complete in helping. This corroborates Cryder, Loewenstein and Seltman’s (2013) research that helping behaviour can lead to feeling of satisfaction within the helper. In helping behaviour, individuals can be moved by personal goals or achieve the satisfaction they have after being able to solve problems. In this case, altruistic motivation and egoism are not a dichotomy but interrelated things (Krebs 1991). In line with research conducted by Hogg and Vaughan (2002) and Baron and Byrne (2005) who state that helping behaviour is an act that provides more benefits for others who are helped, and there are also benefits for the person helping.

*Gotong royong* is an altruistic behaviour that is classified as an important psychosocial support in disaster situations. *Gotong royong* is often in the form of a series of community assistance provided for the purpose of support to parties who are in limited or distressed conditions in the community, both in material and non-material terms in order to be able to regain empowerment. In accordance with the psychosocial definition of Smet (1994) which states four dimensions of social support, namely: (1) Emotional support, in the form of expressions of feelings of care, empathy and concern for other individuals; (2) Appreciation support, in the form of positive expressions as a form of respect for other individuals; (3) Instrumental support, in the form of direct material assistance and (4) Informative support, in the form of providing advice, suggestions or feedback to individuals. This research supports the importance of the overall governance in the process of providing support or assistance to disaster survivors, where psychosocial support is not limited to mental and spiritual support but also how physical and material assistance is channelled in an appropriate manner. When community-based relief organisations work together to care for each other, visit each other and ask how they are doing, these are all forms of psychosocial support in the form of emotional support (Smet 1994). Thus, from this research, it is illustrated that the effects of helping community-based volunteers can make individuals feel better psychologically (Jia, Zhong & Xie 2021). In *gotong royong*, this effect is shared by communities in disaster-prone areas so that any type of disaster threat does not affect existing helping behaviour.

In addition, the results also showed that the respondents in the medium altruistic level category was 62.67%, then the high level was 29.45% and the low category was 7.88%. This profile shows the flexibility and understanding among community-based volunteer members not to be bound by fixed and rigid rules in the structure of *gotong royong* culture. It allows volunteer members to choose in what situation, when and using what they can join together to fill the existing shortcomings according to their capacity and point of view. In the culture of collective society, where the neighbourhood of this research location is the respondent’s extended family, this understanding can explain the diversity of the level of altruistic categories above. According to Deaux and Wrightsman (1984), individuals tend not to help other individuals who need help when there are already many people who have the potential to be helpers of the same action around them. Murata, Imamura and Katoh (2009) mention that individuals must ensure their own safety first before helping others. This is a basic and common principle in safety and emergency conditions. In the context of disasters in disaster-prone areas with Indonesian culture, it is understandable if community-based disaster volunteers apply this rational altruistic behaviour by ensuring the condition of their family is safe and getting their spouse’s approval first before helping. In reviewing the sustainability of the process of helping as a volunteer in accordance with Indonesian culture, namely *gotong royong*, strengthening community support for volunteers is through great acceptance of the situation of limitations and the need for volunteers for the safety of their family members. *Gotong royong* allows for the shortcomings and imperfections of community members to be complemented by other community members.

## Conclusion

Based on the research findings that have been described and discussed, it can be concluded that there is no difference in the altruistic behaviour of community-based natural disaster volunteers with the type of disaster threat. Other findings show that the majority of respondents are in the medium-level altruistic behaviour category, followed by high-level altruistic behaviour and then low-level altruistic behaviour. For disaster governance policymakers, the results of this study can be considered as information in developing programmes by paying attention to and supporting the altruistic behaviour of community-based disaster volunteers. There is no need to doubt the sincerity of volunteers because it is not influenced by the type of disaster threat that exists. In addition, continuous efforts are needed to preserve local wisdom values in disaster risk reduction programmes, especially the dynamics of volunteer altruistic behaviour that is collective in nature, namely, *gotong royong*.
